# Influence of Varying Tensile Stress on Domain Motion

**DOI:** 10.3390/ma15093399

**Published:** 2022-05-09

**Authors:** Kun Zeng, Guiyun Tian, Jia Liu, Bin Gao, Yi Liu, Qianhang Liu

**Affiliations:** 1 School of Automation Engineering, University of Electronic Science and Technology of China, Chengdu 611731, China; liujia617200@163.com (J.L.); bin_gao@uestc.edu.cn (B.G.); liuyi1024@std.uestc.edu.cn (Y.L.); q.h.liu@std.uestc.edu.cn (Q.L.); 2 School of Engineering, Newcastle University, Newcastle upon Tyne NE1 7RU, UK

**Keywords:** varying stress, domain motion, threshold stress value, grain boundary, nondestructive evaluation

## Abstract

Magnetic domain motion has been widely studied in the fields of spintronics, nanowires, and thin films. However, there is a lack of such studies on industrial steels, especially for domain motion under the action of varying stress. Understanding domain motion under stress is helpful for the improvement of evaluation accuracy and the establishment of theoretical models of passive, nondestructive testing technology. This paper presents the influence of varying tensile stresses on the magnetic domain motion of silicon steel sheets. Magnetic domain rotation and domain wall displacement were characterized using magnetic domain images, and their motion mechanisms under elastic and plastic stresses are presented. The results show that the domain rotation under stress involves reversible and irreversible changes. The effect of material rearrangement on domain rotation and domain wall displacement after plastic deformation is discussed. Based on the motion mechanism, a threshold stress value (*TSV*) required for the complete disappearance of the supplementary domains in the elastic range is proposed, enabling the classification of the elastic stress ranges in which the reversible and irreversible domain rotations occur. In addition, the effect of microstructure on TSV is also discussed, and the results show that the regions far away from the grain boundary need larger stresses to complete an irreversible domain rotation. Additionally, the domain width and orientation also affect the *TSV*. These findings regarding the domain motion mechanism and *TSV* can help to explain the sequence of domain rotation under stress and modify the stress assessment under dynamic loads in electromagnetic nondestructive evaluation, especially in the magnetic memory method.

## 1. Introduction

Electromagnetic nondestructive testing of metallic components is generally performed based on the coupling of stress and magnetization [1]. Liu et al. [2] used a multifunctional sensor to simultaneously measure magnetic Barkhausen noise (MBN), tangential magnetic field, and hysteresis lines for tensile steel bars with different surface hardening layer depths. The proposed method can achieve an average prediction error of less than 5.3% for tensile stress. The magnetic recording method (MRM) was used for monitoring ferromagnetic components after magnetization by Tomasz Chady et al. [3]. The study results showed that the signal increased linearly in the elastic range and changed abruptly around the time the yield strength was reached. Ding et al. [4] discussed the effect of the effective damping of the magnetized wall motion on the asymmetric shape of the MBN statistical distribution function under varying stresses, and they proposed skewness as a new feature for determining the applied stresses. Fagan et al. [5] analyzed the MBN energy hysteresis cycle and applied this method to two ferromagnetic materials with significantly different behaviors under uniaxial tensile stress. Bajracharya et al. [6] investigated the effects of stress variations in steel plates using eddy current detection methods. As a complement and extension, many scholars have also investigated the connections between stress, macroscopic magnetic parameters, and microstructure. Qiu et al. [7] proposed a threshold magnetic field to characterize the dynamic behaviors of magnetic domains in different grains under stress and magnetic fields, and the results showed that tensile stress blocked the movement of 180° magnetic domains. Liu et al. [8,9] bridged the gap between magnetic domain motion and MBN. They quantified the difference in the distribution of magnetic domains caused by the increase in magnetoelastic energy at different positions with different tensile stresses. Betz et al. [10] deduced the relationship between grain orientation, corresponding volume, and supplementary domain structure in high-permeability steels. Cao et al. [11] measured the residual stresses in nonoriented silicon steel after stamping and observed the magnetic domains using the Bitter method. In their study, the unannealed samples were found to exhibit complex magnetic domain patterns with varying domain widths. Qiu et al. [12] investigated the effect of magnetic-field and stress-induced domain reorientation on MBN, revealing the complex domain alignment process in transverse electrical steels. The difference in the mechanism of magnetic domain refinement between quasi-static and dynamic processes was described by Schäfer et al. [13]. The role of grain boundaries and mechanical stress on magnetic domain formation and flux propagation was also discussed by them. Perevertov et al. [14] studied the changes in domain structure in the mechanical stress field, and the results showed that under compressive stress, the 180° magnetic domains rotate in the 90° direction, eventually forming transverse domains. However, since it is difficult to capture real-time microstructural features accurately, these research works mainly focused on the microstructures under a specific stress status during the magnetization process. Moreover, the role of continuous varying stress has not been discussed separately.

Compared to the above active detection techniques, the magnetic memory technique (MMM), which has significant application prospects in stress assessment, relies only on the excitation of the geomagnetic field, demonstrating the magnetization changes caused by stress. Gao et al. [15] performed measurements of the stress-induced surface magnetic field intensity *H_p_*(*y*) on low-carbon steel plate specimens. Roskosz et al. [16] evaluated residual stress in ferromagnetic steels based on residual magnetic field (RMF) measurements, and various discussions on the application of RMF have been provided in their subsequent studies. Bao et al. [17,18] discussed the effects of sample size and temperature on MMM, suggesting that they affect the magnitude of the residual magnetic field rather than changing the magnetic curve. The theoretical modeling of stress-induced changes in magnetic properties parameters from the dual-dipole model and the magneto-elastoplastic coupling model was also carried out by Huang [19] and Shi [20]. However, the research was mainly based on the phenomenology theory of macroscopic stress and macroscopic magnetic field, which fails to establish the correspondence between domain motion and the macroscopic magnetic memory signals. At the same time, the spatial properties of transient features have often been neglected.

In summary, the study of the influence of continuous varying stress on magnetic domain motion is an extension of the stress-induced domain motion in thin films [21,22], nanomaterials [23,24], and other fields [25]. This study also contributes to the explanation of the relationship between dynamic loads and magnetic domain motion in nondestructive testing technology from a microscopic perspective. This paper aimed to study the influence of varying stress on domain motion. Magnetic domain images under varying tensile stress along the rolling direction were captured in real-time with the help of a magneto-optical Kerr microscopy system (MOKE). Magnetic domain rotation and domain wall displacement were characterized using magnetic domain images, and the motion behaviors under elastic and plastic stresses are discussed. Based on the motion mechanism, the threshold stress value (*TSV*) required for the complete disappearance of the supplementary domains is proposed, and the influential factors are discussed. The article is structured as follows: After the introduction, the mechanism of domain motion and the characterization method are discussed. Subsequently, the domain motion under continuously varying stress is demonstrated, and, based on the domain motion, the *TSV* and the influential factors are proposed. Finally, conclusions and prospects for future work are presented.

## 2. Methodology

### 2.1. Domain Image Sensing and Domain Motion Characterization

**Figure 1:**
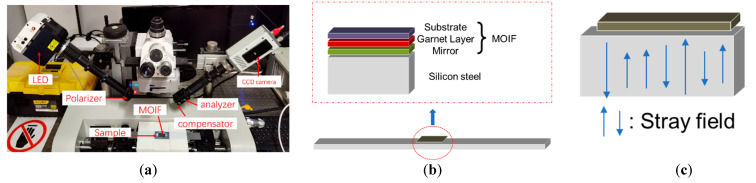
The principle of magnetic domain sensing using the hybrid system of MOKE and MOIF. (**a**) is the hybrid system of MOKE and MOIF, (**b**) is the structure of MOIF, and (**c**) is the principle of stray field mapping to MOIF.

When a beam of monochromatically polarized light shines on the surface of a magnetic material, the polarization plane of the reflected light has a rotated angle compared to the polarization plane of the incident light. This angle is called the magneto-optical Kerr rotation angle. A magnetic domain image can eventually be formed by converting the Kerr rotation angles into the corresponding intensity values in MOKE. The intensity of each pixel of the magnetic domain image is proportional to the magnetization intensity. However, a high degree of surface polishing is required to obtain the Kerr rotation angle. This shortcoming can be overcome with the aid of magneto-optical indicator films (MOIF), as shown in Figure 1a. With this hybrid system of MOKE and MOIF, the magnetic domains of unpolished samples can be easily observed in real-time [26]. The MOIF used in this paper displays perpendicular anisotropy with a large Verdet constant. As shown in Figure 1b, it consists of a three-layer structure: a substrate layer, a garnet film layer, and a reflective mirror layer. This MOIF is very sensitive to mapping the perpendicular component of the stray field of the ferromagnetic material below, which indirectly generates the rotation angle and produces domain images in MOKE. Due to the different intensities and orientations of the stray fields at different positions (Figure 1c), the rotation angle varies, showing a bright and dark magnetic domain texture structure.

**Figure 2:**
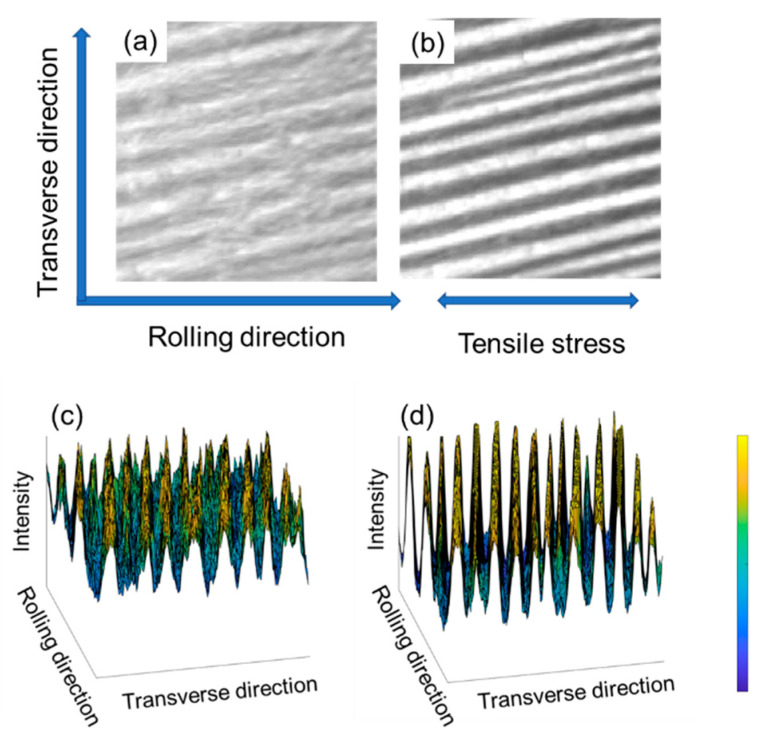
Magnetic domain images in 2D view and 3D view. (**a**,**c**) are views without applied stress, (**b**,**d**) are viewed with 180 MPa applied stress.

Typical magnetic domain images of silicon steel obtained by the MOKE are shown in Figure 2a,b. It can be seen that the magnetic domain images changed significantly after the stress was applied. When the contrast between the light and dark areas is more pronounced, the striped domain wall information is displayed more prominently. The changes in intensity in the corresponding 3D views of Figure 2c,d show this change more clearly. 

The intensity here can be regarded as the magnetization intensity of the underlying sample or the intensity of the domain image because they are proportional to each other. Within the same magnetic domain, the direction of the intensity is approximately the same, with only a very few supplementary domains differing in orientation. The intensities of two adjacent magnetic domains show a transition at the domain walls. The difference in intensity at different locations is not significant in the unstressed state, although it exists. After applying stress, the directions of the intensities within the domains do not change, but the magnitudes increase significantly due to the domain rotation. The domain walls, which are not significantly differentiated in the unstressed state, become more pronounced after applying stress, and their positions are shifted somewhat.

In summary, the magnitude and distribution of intensity can be used to characterize the domain rotation and domain wall displacement before and after tensile stress is applied. Since the domain size is too small for accurate tracking and analysis of a single domain, statistical analysis of multiple domains in a region is used to describe its motion behaviors better, and the size of the region is given as m×n pixel units. Moreover, the domain rotation and domain wall displacement are characterized using the magnetic domain image corresponding to this region. In the case of magnetic domain rotation, this can be expressed as a normalized relative intensity change, *MV*, which is

(1) $$MV=\frac{I-I_{0}}{I_{max}-I_{0}}$$

(2) $$I=\frac{\sum_{i=1}^{m}\sum_{j=1}^{n}I\left(i,j\right)}{mn}$$

where $I\left(i,j\right)$ denotes the intensity corresponding to the point with coordinates $\left(i,j\right)$ on the magnetic domain image, *I* denote the average intensity value of the domain images, $I_{0}$ denotes the average intensity value of the domain image before any stress is applied, and $I_{max}$ is the maximum *I* value in all domain images. According to [27], the relationship between the normal component of stray field B and the intensity of the magnetic domain image I can be given as B∝I. Therefore, the intensity at each point in the magnetic domain image can also correspond to the degree of the magnetization vector rotation at that location. By obtaining the *MV* parameters of a specific magnetic domain image, it is possible to know the relative rotation at this time.

The magnetic domain wall displacement can be expressed in normalized relative angular second-order moment changes (*ASM*). In the Gray-level co-occurrence matrix (GLCM), the *ASM* is also referred to as energy and reflects the degree of uniformity of intensity distribution and texture coarseness. If there is a displacement of the magnetic domain walls, then the distribution of the domain images intensity will change, resulting in a change in the *ASM* value; that is,

(3) $$ASM=\frac{II-II_{0}}{II_{max}-II_{0}}$$

(4) $$II=\frac{\sum_{i=1}^{m}\sum_{j=1}^{n}I^{2}\left(i,j\right)}{mn}$$

where II denotes the average angular second-order moment value of the domain images, $II_{0}$ denotes the average angular second-order moment value of the domain image before any stress is applied, and $II_{max}$ is the maximum *II* value in all domain images. The variation in *MV* and *ASM* allows the quantitative characterization of the magnetic domain rotation and domain wall displacement under stress to study the mechanism of domain motion.

### 2.2. Mechanism of Domain Motion and the Threshold Stress Value

The magnetic domains result from the interaction between the magnetic moments within the crystal that eventually form the system with minimum energy. The total energy $E_{total}$ is composed of magnetic exchange energy, anisotropy energy, magnetoelastic energy, etc. The magnetic exchange energy and anisotropy energy are given by Equations (5) and (6).

(5) $$E_{ex}=-\underset{i\neq j}{\sum }J_{ij}S_{i}\cdot S_{j}$$

(6) $$E_{a}=K_{0}\left(cos^{2}\theta_{1}cos^{2}\theta_{2}+cos^{2}\theta_{1}cos^{2}\theta_{3}+cos^{2}\theta_{2}cos^{2}\theta_{3}\right)$$

where *i*, *j* denote the spin moments of different electrons, $J_{ij}$ denotes the coefficient of exchange integration, $K_{0}$ denotes the magnetic anisotropy constant, and $\theta_{1},\theta_{2},\theta_{3}$ denotes the angle between the magnetization vector and the x, y, and z axes.

$J_{ij}$ is positive in ferromagnetic materials. When the magnetization vectors are parallel to each other, the magnetic exchange energy is weaker; when they are antiparallel, the magnetic exchange energy is stronger. As shown in Figure 2c,d, the direction of the magnetic moments does not change suddenly in the adjacent zones, but there is a transition zone. Theoretically, a more expansive transition zone leads to smaller magnetic exchange energy. However, the spin direction of the electrons is easily shifted. Thus, the anisotropy energy in the transition zone is higher than in the interior domain zone, and the narrow transition zone helps reduce the number of spins without an easy axis. This transition zone is the so-called magnetic domain wall, and the contradictory requirements of magnetic exchange energy and anisotropy energy give the domain wall a well-defined and complex structure.

The mechanism of magnetic domains is domain rotation and domain wall displacement. Current research on the mechanism of domain motion is focused on the mechanisms of domain rotation and domain wall displacement towards the direction of the external magnetic field. This process, also known as technical magnetization, is usually divided into three stages: reversible domain wall displacement in a weak magnetic field, irreversible domain wall displacement in a moderate magnetic field range, and reversible domain rotation in a strong magnetic field, which tends towards saturation as the magnetic field gradually increases. 

**Figure 3:**
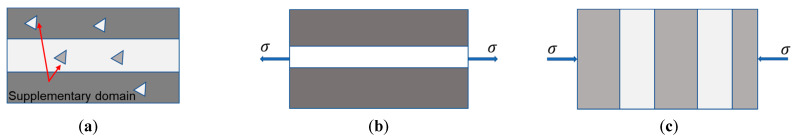
Domain transformation caused by stress. (**a**) Initial state without stress, (**b**) with tensile stress, and (**c**) with compressive stress.

The magnetoelastic energy is the basis for the magnetic domain motion caused by stress and can be expressed as

(7) $$E_{\sigma }=-\frac{3}{2}\lambda_{s}\sigma cos^{2}\theta$$

where $\lambda_{s}$ is the saturation magnetostriction coefficient, σ is the applied stress or strain, and θ is the angle between the magnetization vector and the stress direction. For positive magnetostrictive materials ($\lambda_{s}$ > 0), the tensile stress causes the angle θ to decrease and the compressive stress causes the angle θ to increase. In the stress-free case, the 180° magnetic domains are predominant in silicon steel, thus showing a distribution of striped domains. However, as shown in Figure 3a, there are still supplementary domains and the magnetic moments are not in the same direction as the striped domain. In Figure 3b, the magnetization vector rotates in the direction of applied tensile stress, resulting in the disappearance of the supplementary domain. At the same time, the domain wall displacement is generated under stress. In Figure 3c, the magnetic domain vector is rotated in the direction perpendicular to the stress after the application of compressive stress, resulting in the transformation of the striped domain into the transverse domain (90° domain).

The three energies mentioned above influence domain rotation and domain wall displacement. However, another energy, the magnetostatic energy, is the most fundamental factor in forming the magnetic domain structure. Magnetostatic energy originates from the mutual balancing effect of magnetic moments in ferromagnetic materials. It effectively reduces the overall magnetic moment of the system and is also known as demagnetization energy. The energy can be expressed as

(8) $$E_{s}=-\mu_{0}\int H_{d}dM_{s}=\frac{\mu_{0}}{2}N_{d}\cdot M_{s}^{2}$$

where $H_{d}$ denotes the demagnetization field, $M_{s}$ is the spontaneous magnetization within the magnetic domain, $N_{d}$ is the demagnetization factor, and $\mu_{0}$ is the magnetic permeability.

The presented study mainly focused on the mechanism of domain motion caused by continuously varying stress. After tensile stress was removed, three types of residual stresses existed in the material. Type I is the macroscopic residual stress, while Type II and III are the microscopic residual stresses. The distribution of residual stresses was exceptionally heterogeneous in different grains, even in different regions within the same grain, causing local anisotropy in the material and thus creating a complex magnetic domain structure and changing the domain motion characteristics. Therefore, the mechanism of magnetic domain motion in different grains was also proposed by considering the grain boundary, domain width, and domain orientation.

### 2.3. Experimental Setup

A total of 20 silicon steel sheets, labeled 23Z110 type, with a yield strength of approximately 320 MPa and dimensions of 0.23 mm×30 mm×300 mm $\left(\mathrm{thickness}\times \mathrm{width}\times \mathrm{length}\right)$, were used in this study. The chemical components of the silicon steel are shown in Table 1. The applied tensile stress was loaded and unloaded at a rate of 0.5 mm/min and was controlled by a specially designed mini-stretch machine, which ensured the unchanged position of the observed area. Above the sample, and in situ MOKE with a frame rate of 16 was used for real-time magnetic domain image acquisition. All processes were performed at constant environmental temperature and humidity.

## 3. Result and Discussion

A silicon steel sheet named Sample 1, with large grains, was used as an example to demonstrate the mechanism of magnetic domain motion caused by continuously varying tensile stress. Next, the *TSV* of the irreversible domain rotation was proposed, and further, the relationship between *TSV* and influential factors was discussed.

### 3.1. Mechanism of Domain Motion in the Elastic and Plastic Range

**Figure 4:**
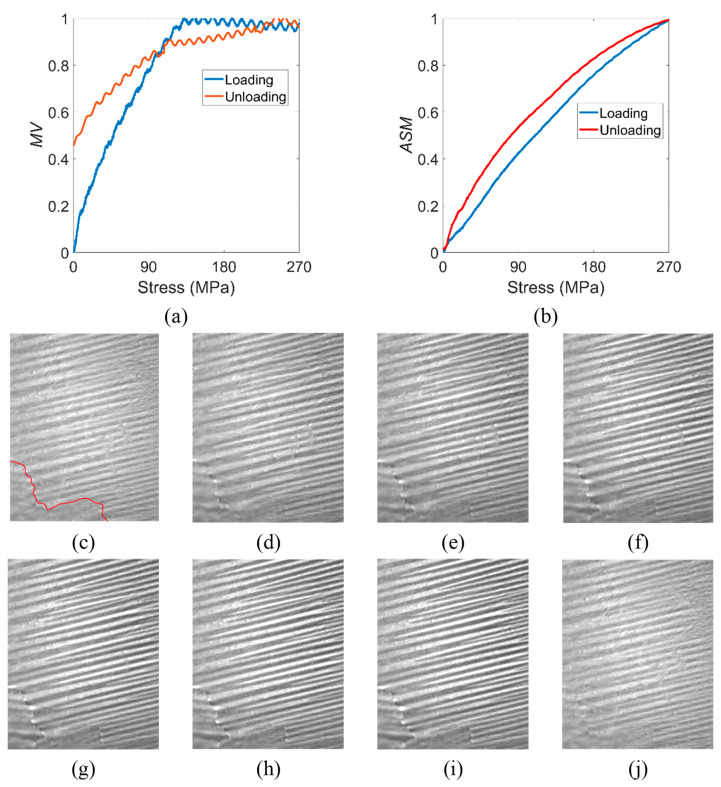
Variation in characterization parameters (**a**) *MV* and (**b**) *ASM* during the loading and unloading process in the elastic range, and the corresponding domain images of (**c**) initial 0 MPa, (**d**) 45 MPa, (**e**) 90 MPa, (**f**) 135 MPa, (**g**) 180 MPa, (**h**) 225 MPa, and (**i**) 270 MPa applied stress and (**j**) after 270 MPa stress was removed. The red line in (**c**) represents the position of the grain boundary. The size of the domain image is 12 mm × 15 mm.

The variations in the characterization parameters *MV* and *ASM* during the loading and unloading process of 270 MPa tensile stress are shown in Figure 4. The corresponding domain images at different stress statuses are also listed. The quantitative analysis showed that *MV* changed linearly with stress in the low-stress field (about 0–120 MPa) during the loading process. However, after the stress exceeded 120 MPa, *MV* no longer increased linearly but entered an oscillatory steady state. In contrast, the *ASM* was linearly related to the increasing stress applied throughout the loading process. The magnetic domain images also show this variation in magnetic domain rotation and domain wall displacement in the elastic range. As shown in Figure 4c–f, the contrast of the adjacent magnetic domains increased significantly and the domains became narrower as the stress increased. However, as shown in Figure 4g–i, no significant change existed in the contrast of the adjacent domains, but it can be seen that the width of the domains continued to decrease. 

In addition, the *MV* showed two trends during the stress unloading process. During the applied stress being unloaded from 270 MPa to 100 MPa, *MV* showed a linearly decreasing trend. After the stress dropped below 100 MPa, the *MV* fell with a more significant linear trend until the applied stress reached zero. It is noteworthy that the *MV* did not return to the zero value at this time, and there was still a non-negligible change compared with the initial state. On the other hand, the *ASM* maintained a hysteresis variation throughout the unloading process with a slightly larger value than the loading process. Eventually, the *ASM* was almost the same as the initial state after the stress was removed.

The characterization parameters were related to the magnetic domain motion. The wall energy of the supplementary domain inside the striped domain was relatively small. Under stress, the domain wall between the supplementary domain and the striped domain moved significantly, causing the area of the supplementary domain to decrease and the magnetization vector of the supplementary domain to rotate in the direction of the striped domain. *MV* was affected by the rotation of both the supplementary and striped domains. When the stress reached a specific value, the supplementary domains disappear entirely and completed the merging process with the striped domain. However, the domain wall energy between adjacent striped domains was large, and the elastic stress only caused a tiny reversible domain wall displacement, as well as a small fraction of the magnetization vector inside the striped domain to rotate. At this time, the *MV* was only affected by the rotation of the striped domain. Therefore, when the stress reached a specific value, significant domain rotation no longer occurred even if the elastic stress continued to increase. Although there were also domain walls between the supplementary domains and the striped domains, their thicknesses and domain wall energies were much smaller than those of the domain walls between the striped domains. Therefore, the reorganization process of the supplementary domains and the striped domains had almost no effect on the overall domain wall displacement. Therefore, the influence on the *ASM* parameters was mainly on the domain walls between the striped domains.

After the elastic stress was removed, the domain walls of the striped domain returned to their initial positions, and the magnetization vector within the striped domain also returned to its initial angle. However, the merging with the supplementary domains meant that the striped domains could not wholly rotate to their initial states.

**Figure 5:**
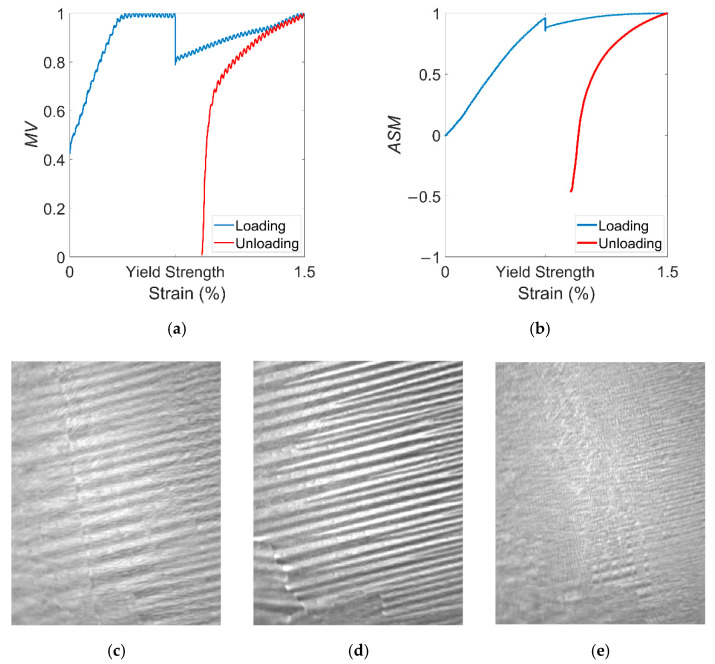
Variation in characterization parameters (**a**) MV and (**b**) ASM during the loading and unloading process in the plastic range and the corresponding domain images (**c**) initial 0 MPa, (**d**) with 1% strain and (**e**) after 1% strain was removed. The size of the domain image is 12 mm × 15 mm.

Next, the magnetic domain motion was extended to the continuously varying plastic range stress. The 1% strain loading and unloading process was performed on the sample that had just unloaded the 270 MPa stress. The variation in the characterization parameters *MV* and *ASM* throughout the process and their corresponding magnetic domain images are shown in Figure 5. It can be seen that there was a sudden drop in both parameters when the yield strength was exceeded. After that, both parameters continued to increase linearly with strain, almost reaching their pre-plastic deformation values at 1% strain. The difference between the magnetic domain pattern at this point (Figure 4j) and the one in the elastic range (Figure 5d) was not significant. After the stress exceeded the yield strength, the nonuniform plastic deformation of the material caused microscopic stresses and grain boundary migration. This inhomogeneity of plastic deformation between grains was the main cause of microstructure evolution. On the other hand, plastic deformation increased the number of dislocations and created pinned energy centers. The balance between the magnetic domain energy was broken, and the striped domain remained under the action of magnetoelastic energy.

After the start of the unloading process, the two parameters decreased linearly. When the strain dropped to about 0.61%, the two parameters started to decrease drastically until they reached a minimum value at 0.59% residual strain. At this point, the *MV* was similar to the initial value, while the *ASM* became negative. At this point, the magnetic domain image (Figure 5e) shows that after 1% strain was removed, the magnetic domain was dominated by transverse domains, with only some striped domains still present near the grain boundaries. Since the strain was applied at both ends of the sample, it is ideal to assume that the deformation was uniformly distributed. After the yield strength was reached, some of the work done by the strain was converted into thermal energy, and a small portion was stored as distortion energy. The anisotropy energy and magnetic exchange energy also changed, resulting in transient changes in domain rotation and domain wall displacement at the yield strength. The distortion energy also continued to increase with strain, resulting in a lesser degree of domain motion in the plastic range than in the elastic range. When the strain dropped to 0.61%, the sample was subjected to macroscopic stress in the direction of the compressive stress. Due to the change of magnetoelastic energy caused by the compressive stress, the domain rotated rapidly in the direction of 90° and the domain walls also changed, resulting in the formation of mainly transverse domains. Therefore, in contrast to the domain motion after elastic stress, the magnetic domain after plastic deformation showed a two-stage change: the recovery of the initial position and the reorganization of the new domain. After unloading, residual strain existed, and distortion energy existed in the form of residual compressive stresses. Under compressive stresses, the domain wall shifted from the rolling direction to the transverse direction, as well as the domain rotation changed in the opposite direction. The grain boundary hindered the dislocation mobility, which increased the deformation resistance so that some of the striped domains were still present at the grain boundary.

The Jiles model [28] and subsequent studies were widely applied to illustrate stress-induced magnetization. The domain motion mechanism under varying tensile stress in this paper was used for comparison with the magneto-mechanical constitutive relation model under dynamic loading proposed by Shi et al. [29,30]. The magnetic domain motion, especially the domain motion during the elastic stress loading and unloading process, validated the theoretical model and is important for studying the relationship between varying stress and surface micromagnetic signals from a microscopic perspective.

### 3.2. The Proposed TSV and the Influential Factors

**Figure 6:**
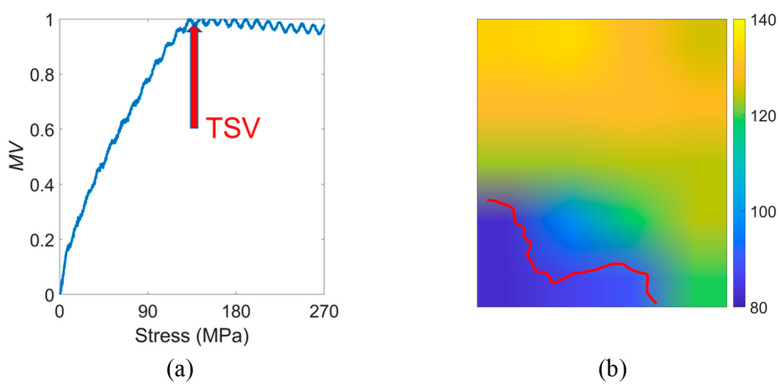
(**a**) Proposed TSV and (**b**) the distribution in Sample 1.

The stress required for the complete disappearance of the supplementary domains is called the ‘threshold stress value’ (*TSV*) and is shown in Figure 6a. The *TSV* divides the domain rotation of the material into reversible and irreversible phases. As can be seen in Figure 2, the magnetic domain rotation and the magnitude of the RMF are closely related. From the macroscopic magnetic parameter perspective, the residual magnetic field signal increases with stress, but after reaching the *TSV*, the magnitude of the residual magnetic field no longer rises [17].

In Section 3.1, it is mentioned that for Sample 1 as a whole, the *TSV* was 120 MPa. However, it can also be seen from the magnetic domain images that the domain pattern was different in different regions, especially at the grain boundaries, which had a very different distribution of domains at the grain interior. Therefore, the study of *TSV* at the grain boundaries and inside grains is discussed below.

The *TSV* for different regions in Sample 1 was captured using a scanning window with a size of 10 mm × 10 mm, and the distribution is shown in Figure 6b. The corresponding *TSV* values are displayed in the color bars. It can be seen that the distribution of *TSV* showed a clear regional difference. In the bottom left part near the grain boundary, which is represented by a red line, the *TSV* values were distributed in the range of 80–100 MPa. The middle part of the sample, which was not far from the grain boundary, had *TSV* values of 100–120 MPa. The highest *TSV* values were found in the upper part of the sample, which was far from the grain boundary, at about 120–140 MPa.

**Figure 7:**
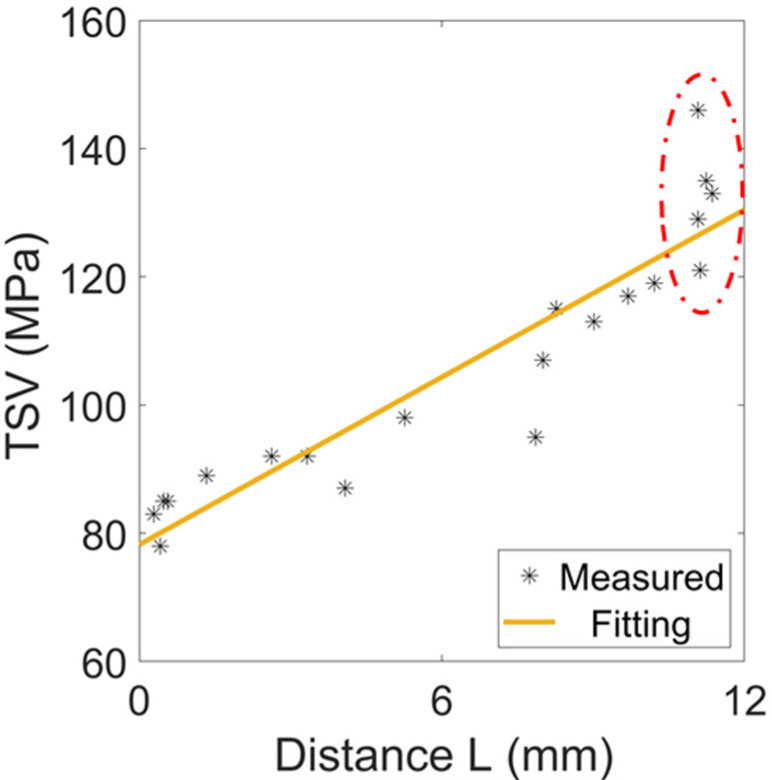
The relationship between *TSV* and the distance *L* to the nearest grain boundaries in different samples.

It can be seen that the magnetic domain rotation is closely related to the location of grain boundaries. Next, 20 samples with different grain sizes were analyzed for *TSV*, and the characterization areas were randomly selected with a size of $10\;\mathrm{mm}\times 10\;\mathrm{mm}\;\left(\mathrm{width}\times \mathrm{length}\right)$. The characterized areas had different distances from the grain boundary, and the distance *L* was defined as the distance from the center of the observed area to the nearest grain boundary. The relationship between *TSV* and *L* in different samples is shown in Figure 7. The distribution of *TSV* and the fitted curves show that the regions farther away from the grain boundary had a larger *TSV* and their magnetic domain rotation was less hindered. The magnetic domains at the grain boundaries were subject to internal stresses caused by crystal defects such as dislocations, hindering domain rotation, and domain wall displacement. In addition, those crystal defects also led to a reduction in the domain wall area. Since the total domain wall energy is related to the domain wall area, the domains at the grain boundaries had a relatively small domain wall energy and they were more stable than the domains inside the grains. As a result, the irreversible components of the domain motion at the grain boundaries were relatively more minor and *TSV* was more easily achieved.

The relationship between the distance *L* to the grain boundary and *TSV* were statistically analyzed for 20 samples, and the Pearson correlation coefficient was found to be 0.927. However, it is worth noting that the samples with similar distances to the nearest grain boundary, for example, the five samples circled by the red ellipse in Figure 7, still had significant differences in *TSV*. If these five samples were excluded from the statistical analysis, the Pearson correlation coefficient between *L* and *TSV* of the other samples reached 0.978. The Pearson correlation coefficient showed that the distance *L* to the grain boundary is the most important factor influencing the saturation of the magnetic domain rotation. Therefore, other factors besides the grain boundary location can affect the *TSV*.

The magnetic domains of the five samples in the red ellipse are listed in Table 2 in the order of smallest to largest *TSV*. The distances to the grain boundary of those five samples were similar, but the domain widths and orientations were different. Obviously, the wider the magnetic domains of the sample, the larger the *TSV* value. As can be seen in Equation (8), to ensure the minimization of magnetostatic energy, the presence of more magnetic domains is required for a region of fixed size, which leads to a decrease in the domain width. It is assumed that the striped domains have the same domain width $w_{1}$ in a region of width m. At this point, the relationship between the magnetostatic energy $E_{s1}$ of a single domain and the total magnetostatic energy $E_{s}$ of the region is

(9) $$E_{s1}=\frac{E_{s}w_{1}}{m}$$

Assume that in the same region, there are also striped magnetic domains with domain width $w_{2}$, and that the magnetostatic energy of these domains is

(10) $$E_{s2}=\frac{E_{s}w_{2}}{m}$$

Thus, the difference $∆E_{s\_12}$ of the magnetostatic energy in striped magnetic domains with different widths can be expressed as

(11) $$∆E_{s\_12}=\frac{E_{s}(w_{1}-w_{2})}{m}$$

The difference in magnetostatic energy in magnetic domains of different widths is proportional to the domain width, with other influencing factors being the same. A decrease in magnetostatic energy leads to an increase in magnetic exchange energy so that the magnetic exchange energy increases linearly with the decrease in the domain width. Since the magnetic exchange energy is isotropic, the magnetization vector inside the domain is already rotated in the direction of the easy magnetization axis in the state of the degenerate magnetic field. Therefore, the smaller width of the domain has a smaller area of additional domains; as a result, lower energy and lower stress are required to complete the rotation of the magnetization vector in the 180° direction. Moreover, since the change in magnetic exchange energy is proportional to the change in domain width, the *TSV* is theoretically linearly related to the domain width for domains with the same distance *L* to grain boundaries and domain orientation. 

**Figure 8:**
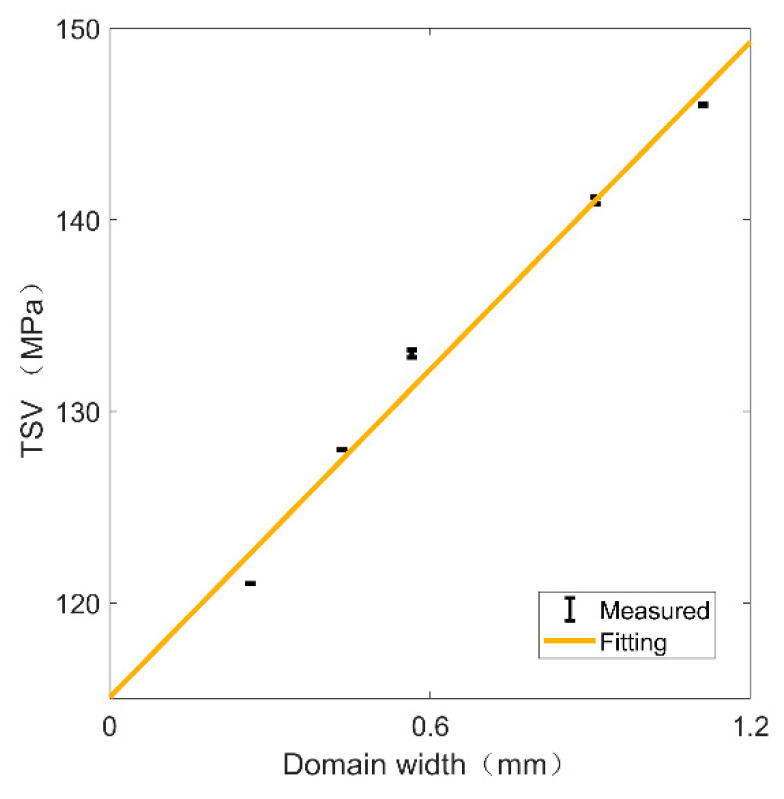
Correlation of TSV and domain width of samples with similar distance L to the grain boundary. The error bars show the effect of domain orientation.

The relationship between domain width and *TSV* is also shown in Figure 8 for different samples with similar distances *L* to the grain boundary. According to Equation (7), the angles between the stress and the domain orientation were also taken into consideration, and are presented in the form of an error bar plotted in the figure. It can be seen that with the same distance to the grain boundary, the magnetic domain width was approximately linearly correlated with the TSV, and the Pearson correlation coefficient between those two parameters was 0.99. Considering the domain orientation, the TSV of samples with a similar distance *L* to the grain boundary was still mainly influenced by the domain width. At the same time, the influence of the magnetic domain orientation was relatively tiny.

According to the presented results of the *TSV* for different identical parts, it can be seen that the grain boundary is the most significant influential factor in the *TSV.* For regions with the same distance to the grain boundary, the domain width and orientation also affect the *TSV*.

## 4. Conclusions and Future Work

In this paper, the influence of continuously varying tensile stress on domain motion was investigated by means of real-time magnetic domain images acquired using a hybrid MOKE and MOIF system. The TSV required for a magnetic domain to complete irreversible rotation is proposed, and the influential factors were summarized by exploring different samples. Our results led us to the following conclusions:

(1) Real-time tracking of magnetic domain motion shows the continuation of the magnetic domain wall displacement during the elastic stress loading process, together with the irreversible domain rotation of the supplementary domain, will be complete after specific elastic stress, after which only the reversible domain rotation will exist in the striped domain. After the stress is removed, due to the fusion of supplementary and striped domains, the overall magnetic domain cannot rotate to its initial state, while the domain walls’ displacement is reversible. For domain motion in the plastic range, the presence of distortion energy leads to less intense domain motion behavior during stress loading than in the elastic range. Moreover, the domain pattern changes from striped domains to transverse domains after the applied strain is removed. The domain motion mechanism matches the magneto-mechanical constitutive relation model.

(2) The threshold stress value is proposed to characterize the stress required to complete the irreversible rotation of the supplementary domain in the elastic range. By studying different samples, it was shown that the *TSV* is related to the spatial factors of the microstructures. Specifically, in regions with large distances from the grain boundaries, broad magnetic domains require larger *TSV*s to complete irreversible domain rotation.

This paper complements previous studies of the mechanism of magnetic domain motion caused by continuously varying tensile stress under only the excitation of geomagnetic fields, which are easily ignored in electromagnetic nondestructive testing techniques. The domain motion mechanism validates the magneto-mechanical constitutive relation from a microscopic perspective. Moreover, the proposed *TSV*, together with the influential factors, explains the sequential problem and the irreversible changes in magnetic domain rotation under stress. In particular, the combination of domain motion and macroscopic magnetic parameters can determine the stress range and discriminate the microstructure, which can help with stress assessment in different measured regions using micromagnetic methods, e.g., Magnetic memory method (MMM), magnetic barkhausen noise (MBN). In addition, the quantitative assessment of the influential factors of *TSV*, linking to other magnetic features at micro and marco scale, and its dynamic behaviors [4,8] will be carried out in subsequent studies.

## Figures and Tables

**Table 1:** Chemical components of the silicon steels expressed as weight percentages.

Fe	Si	C	Mn	P	S	Al
Balance	3.00–5.00	0.06	0.15	0.03	0.25	5.10–8.50

**Table 2:** Magnetic domain images and *TSV* of five samples with similar distances *L* to the grain boundaries. The grain boundary of the last sample is outside the right side of the image. The red line represents the grain boundary and the green rectangular box indicates the characterized area.

Domain Images	*TSV*	Distance *L*	Average Domain Width	Domain Orientation
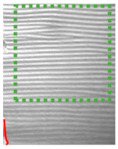	121 MPa	11.08 mm	0.263 mm	0°
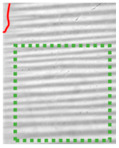	128 MPa	11.13 mm	0.435 mm	0°
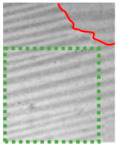	133 MPa	11.24 mm	0.566 mm	11°
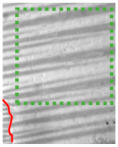	135 MPa	11.36 mm	0.910 mm	10°
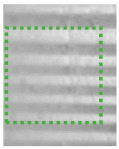	146 MPa	11.08 mm	1.111 mm	−2°
